# Acetaminophen-Induced Liver Injury Exposes Murine IL-22 as Sex-Related Gene Product

**DOI:** 10.3390/ijms221910623

**Published:** 2021-09-30

**Authors:** Hendrik Stülb, Malte Bachmann, Sina Gonther, Heiko Mühl

**Affiliations:** Pharmazentrum Frankfurt/ZAFES, Institute of General Pharmacology and Toxicology, Faculty of Medicine, Goethe-University Frankfurt, D-60590 Frankfurt am Main, Germany; stuelb@med.uni-frankfurt.de (H.S.); M.Bachmann@med.uni-frankfurt.de (M.B.); gonther@em.uni-frankfurt.de (S.G.)

**Keywords:** interleukin-22, inflammation, testosterone, sex, gender, acetaminophen, liver damage

## Abstract

Gaining detailed knowledge about sex-related immunoregulation remains a crucial prerequisite for the development of adequate disease models and therapeutic strategies enabling personalized medicine. Here, the key parameter of the production of cytokines mediating disease resolution was investigated. Among these cytokines, STAT3-activating interleukin (IL)-22 is principally associated with recovery from tissue injury. By investigating paradigmatic acetaminophen-induced liver injury, we demonstrated that IL-22 expression is enhanced in female mice. Increased female IL-22 was confirmed at a cellular level using murine splenocytes stimulated by lipopolysaccharide or αCD3/CD28 to model innate or adaptive immunoactivation. Interestingly, testosterone or dihydrotestosterone reduced IL-22 production by female but not by male splenocytes. Mechanistic studies on PMA/PHA-stimulated T-cell-lymphoma EL-4 cells verified the capability of testosterone/dihydrotestosterone to reduce IL-22 production. Moreover, we demonstrated by chromatin immunoprecipitation that testosterone impairs binding of the aryl hydrocarbon receptor to xenobiotic responsive elements within the murine IL-22 promoter. Overall, female mice undergoing acute liver injury and cultured female splenocytes upon inflammatory activation display increased IL-22. This observation is likely related to the immunosuppressive effects of androgens in males. The data presented concur with more pronounced immunological alertness demonstrable in females, which may relate to the sex-specific course of some immunological disorders.

## 1. Introduction

Interleukin (IL)-22 [1,2] is a leukocyte-derived tissue-protective cytokine [3] of the IL-10 family [4] that, by activating the signal transducer and activator of transcription (STAT)-3 in cells of epithelial origin [5], serves to increase defense, repair, and healing at host environment interfaces. This notable portfolio of IL-22′s biological properties is mediated by, among other factors, enhanced expression of antimicrobial peptides [5,6,7] and antimicrobial [8] inducible nitric oxide synthase [9,10,11,12,13,14], in addition to by increased cellular proliferation and anti-apoptosis [15,16]. Tissue protection by IL-22 is particularly well-established in the case of the liver [17]. Protection by the administration of recombinant IL-22 has been repeatedly verified in models of acute liver injury (ALI) induced by concanavalin A (ConA) [15], lipopolysaccharide/d-galactosamine [18], alcohol [19], or acetaminophen (APAP) [20,21,22]. Endogenous IL-22 production similarly ameliorates ConA-induced ALI [15]. This latter observation indicates IL-22 is a key parameter of the hepatic resolution and repair program [23], which is supposed to determine the course of inflammation and regeneration during ALI.

Immunoactivation can eventually initiate hepatic regeneration by triggering a reparative face of inflammation [23]. This so-called reparative inflammation is mediated, at least partly, by upregulating STAT3-activating cytokines, which have the capability to drive compensatory hepatocyte proliferation during the regeneration phase of ALI [24]. Given its striking potency to act on hepatocytes, IL-22 is supposed to be a key player in that context [17]. APAP-induced ALI, which is paradigmatic for drug-induced ALI, shows extensive necroinflammation that may be pathogenic in early intoxication [25,26] but subsequently initiates regeneration during reparative inflammation [24,27]. Because immune responses are affected by sex in a qualitative and quantitative manner [28,29], it is reasonable to assume that reparative inflammation, and thus the course of ALI, is similarly affected by this parameter. Interestingly, female mice display attenuated APAP toxicity [30,31]. Because the role of sexes in hepatic diseases is a pressing understudied topic, sex-dependent production of pro-regenerative IL-22 was analyzed herein in murine APAP-induced ALI and cell culture models of IL-22 expression.

## 2. Results

### 2.1. Female C57BL/6J Mice Display Increased IL-22 Production Associated with APAP-Induced ALI

**Figure 1:**
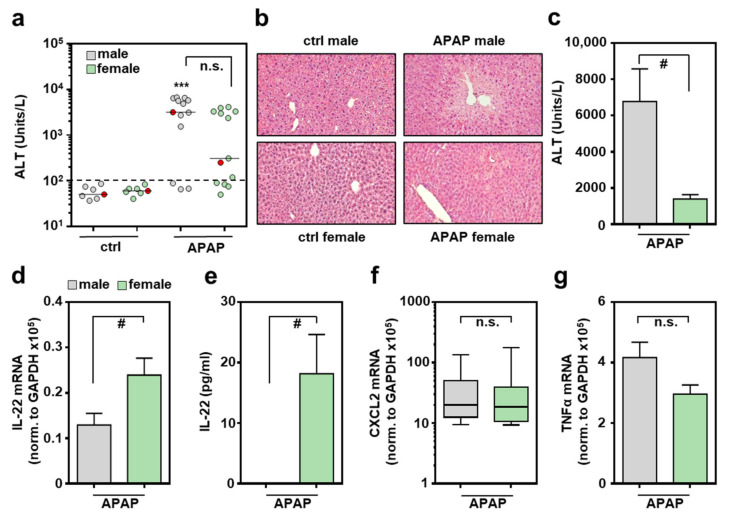
Increased production of IL-22 by female mice undergoing APAP-induced ALI: female and male C57BL/6J mice were treated with either 0.9% NaCl (ctrl) or APAP (300 mg/kg). After 6 h (**a**,**b**,**d**–**g**) or 24 h (**c**), mice were sacrificed and liver tissue and sera were analyzed. Liver damage was determined by serum ALT after 6 h (**a**) or 24 h (**c**) ((**a**): *n* = 7 for ctrl, *n* = 13 for APAP; *** *p* < 0.001 versus ctrl-male; (**c**): *n* = 5; # *p* < 0.05). (**b**) Liver sections (H&E) are shown for representative specimens, labeled red in subfigure (**a**). Hepatic mRNA expression for IL-22 (**d**), CXCL2 (**f**), and TNFα (**g**) was determined by real-time PCR and normalized to GAPDH, and is shown as absolute values (*n* = 10 for APAP-male; *n* = 9 for APAP-female; # *p* < 0.05). (**e**) Serum IL-22 analyzed by ELISA (*n* = 7 for APAP-male, *n* = 8 for APAP-female; # *p* < 0.05). Statistical analysis: (**a**) raw data were analyzed by Kruskal–Wallis with post hoc Dunn’s correction, (**f**) raw data were analyzed by Mann–Whitney-U-test, data are shown as box-plots; (**c**–**e**,**g**) raw data were analyzed by unpaired Student’s *t*-test, data are shown as means ± SEM. n.s., not statistically significant.

IL-22 expression during APAP-induced ALI has been linked to its initial phase at 3 h/6 h, without investigating the role of sexes [32]. Based on these data, male/female mice were exposed to APAP for 6 h. Compared to controls (ctrl), only male mice displayed significantly increased liver damage at this early time point (Figure 1a,b). Both sexes included few mice where APAP failed to induce liver damage at 6 h, and thus displayed serum alanine aminotransferase (ALT) activity in the control range (Figure 1a). This observation is consistent with previously published data [32]. Of note, whereas the 6 h time point only displayed a tendency towards decreased liver damage in female mice, this difference became highly significant at 24 h after APAP administration (Figure 1c). This latter observation is in accordance with previously published data [30,31]. Because necroinflammation driven by liver damage is a prerequisite for induction of inflammatory cytokines in this model, APAP-treated mice without toxicity within 6 h (as defined by ALT levels) were excluded (3 × males, 4 × females; Figure 1a, mice below the dashed line represent a cut-off at 100 units/L of serum ALT) from further analysis when comparing sex-related cytokine responses. Of note, all mice treated with APAP (300 mg/kg) for 24 h developed ALI but also survived this experimental protocol (Figure 1c).

Female mice undergoing APAP-induced ALI displayed significantly enhanced hepatic IL-22 mRNA (Figure 1d), which translated into well-detectable serum IL-22. Notably, all female control sera and all male sera remained IL-22-negative throughout all specimens investigated (Figure 1e). Interestingly, as opposed to the 6 h time point, IL-22 mRNA remained undetectable in the later regeneration phase of APAP-induced ALI, irrespective of the sexes (at 24 h, too low to be validly detectable (*n* = 5 for males and females), data not shown; at 48 h, see reference [32]). In contrast to IL-22, hepatic expression of inflammatory CXCL2 (Figure 1f) and tumor necrosis factor (TNF)-α (Figure 1g) were independent of the respective sex. Because IL-6 has the capability to support IL-22 production [33], hepatic expression of this cytokine was investigated 6 h after administration of APAP. As shown in Supplementary Figure S1a, expression of IL-6 was not significantly affected by the respective murine sex. This also applied to expression of the IL-22-regulating transcription factor aryl hydrocarbon receptor (Ahr) [33] (Supplementary Figure S1b), and to the IL-22-related cytokines [4] IL-20 (Supplementary Figure S1c) and IL-24 (Supplementary Figure S1d). In the case of IL-22R1, we observed a tendency towards increased expression in female mice. However, this interesting tendency did not reach the level of significance in the set of experiments performed (Supplementary Figure S1e).

### 2.2. Increased IL-22 Production by Female Murine Splenocytes after Activation of Innate or Adaptive Immunity In Vitro

**Figure 2:**
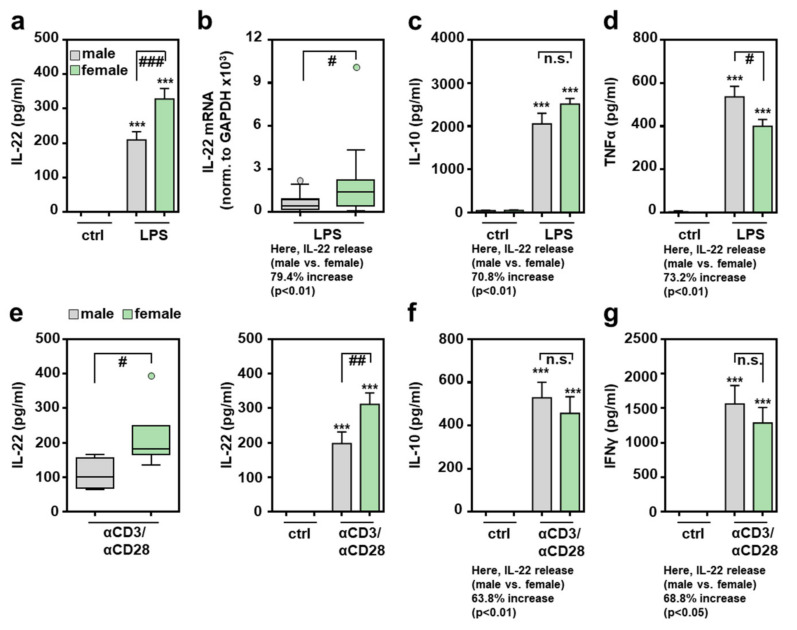
Increased production of IL-22 by female splenocytes exposed to LPS or αCD3/CD28: freshly isolated male and female murine splenocytes of C57BL/6J mice were kept as unstimulated ctrl or stimulated with LPS (1 µg/mL) for 48 h (**a**–**d**) or with agonistic αCD3/CD28 (2 µg/mL, 0.2 µg/mL) for 24 h ((**e**)-left panel) or 48 h ((**e**)-right panel, (**f**,**g**)). Release of the indicated cytokines was determined by ELISA: IL-22 (**a**,**e**), IL-10 (**c**,**f**), TNFα (**d**), and IFNγ (**g**) ((**a**): *n* = 33; (**c**): *n* = 8–14; (**d**): *n* = 13–17; (**e**)-left: *n* = 4–6; (**e**)-right: *n* = 12–16; (**f)**: *n* = 11–13; (**g**): *n* = 8–13; *** *p* < 0.001 versus ctrl of respective sex, # *p* < 0.05, ## *p* < 0.01, ### *p* < 0.001). (**b**) Splenic mRNA expression for IL-22 was determined by real-time PCR and normalized to GAPDH, and is shown as absolute values (*n* = 19; # *p* < 0.05). Statistical analysis: (**a**,**c**,**d**,**e**-right,**f**,**g**) raw data were analyzed by one-way ANOVA with post hoc Bonferroni correction, data are shown as means ± SEM; (**b**,**e**-left) Mann–Whitney-U-test, data are shown as box-plots. n.s., not statistically significant.

Data presented herein demonstrate increased IL-22 production in female mice undergoing acute hepatic necroinflammation. To study this phenomenon in a cellular model resembling a genuine composition of leukocytes, lipopolysaccharide (LPS)/Toll-like receptor-4 (TLR4)-stimulated splenocytes were analyzed. This type of innate immunoactivation was selected because TLR4 serves as an amplifier of danger associated molecular pattern (DAMP)-driven necroinflammation [34] that similarly affects APAP-induced ALI [35,36,37]. Moreover, current knowledge indicates that APAP, by direct action on intestinal stem cells, mediates barrier dysfunction [38], which likely results in modest but operative endotoxemia. Figure 2a,b demonstrates significantly enhanced IL-22 production in LPS-activated female splenocytes. Specifically, IL-22 release by female splenocytes was increased by 56.8% as compared to their male counterparts. IL-22 release from unstimulated cells of either sex was not detectable (Figure 2a). In the same set of samples showing upregulated IL-22 production in female splenocytes, analysis of IL-10 (Figure 2c) or TNFα (Figure 2d) revealed no influence of sex or even reduction of female gene expression. Of note, upregulation of IL-22 expression in female splenocytes was not associated with enhanced expression of Ahr (Supplementary Figure S2a). Interestingly, IL-22-related IL-24 showed a tendency towards increased expression in female splenocytes, although this trend did not reach the level of significance in the set of experiments performed (Supplementary Figure S2b).

Splenic CD4^+^ memory T cells are regarded as a significant source of IL-22 [39]. To directly activate splenic T cells, stimulatory αCD3/CD28 antibodies were used. Figure 2e demonstrates more efficient female IL-22 production after 24 h (left panel) or 48 h (right panel) of T cell receptor activation. Interestingly, αCD3/CD28-mediated induction of IL-10 (Figure 2f) and interferon (IFN)-γ (Figure 2g) did not diverge in relation to the studied sex, demonstrating that more efficient production of IL-22 in female splenocytes is not mediated by generally more efficient cellular activation under the influence of αCD3/CD28.

### 2.3. Androgens Inhibit Splenocyte Production of IL-22

**Figure 3:**
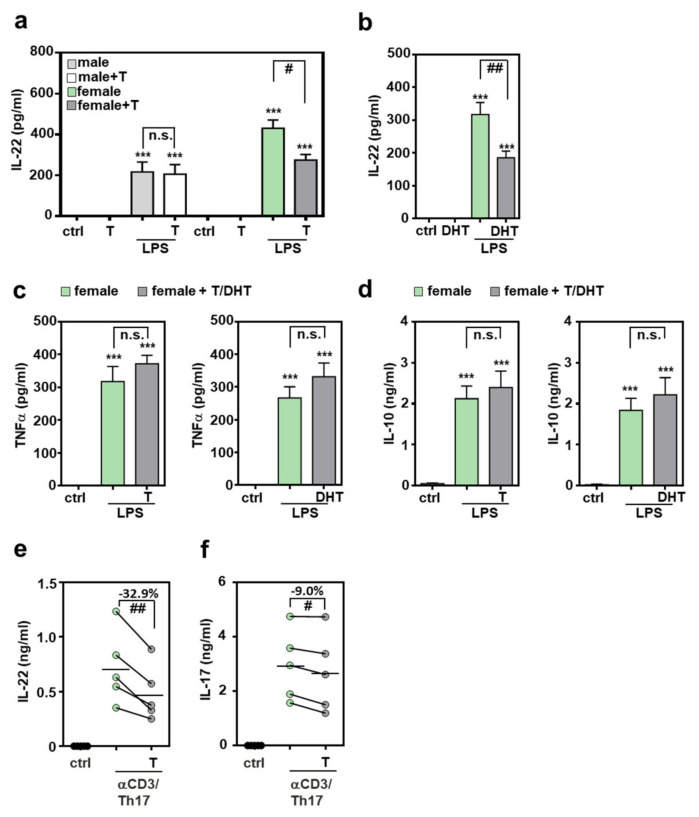
Inhibition of IL-22 production by androgens: (**a**–**d**) freshly isolated male or female murine splenocytes of C57BL/6J mice were kept as unstimulated ctrl or stimulated for 48 h with LPS (1 µg/mL) in presence or absence of testosterone or DHT (both at 1 µM). (**e**,**f**) Splenocytes of female C57BL/6J mice were kept as unstimulated control or cultivated under Th17-differentiation conditions with or without testosterone (1 µM) for 4 days. All cultures were adjusted to a final concentration of 0.0014% (vehicle for testosterone; **a**,**c**-left,**d**-left,**e**,**f**) or 0.029% (vehicle for DHT (**b**,**c**-right,**d**-right)) methanol. Release of indicated cytokines was determined by ELISA: IL-22 (**a**,**b**,**e**), TNFα (**c**), IL-10 (**d**) and IL-17 (**f**) (**a**,**c**-left,**d**-left): *n* = 8; (**b**,**c**-right,**d**-right): *n* = 7; (**e**,**f**): *n* = 5; *** *p* < 0.001 versus ctrl of respected sex, # *p* < 0.05, ## *p* < 0.01). Statistical analysis: (**a**–**d**) raw data were analyzed by one-way ANOVA with post hoc Bonferroni correction; data are shown as means ± SEM; (**e**,**f**) raw data were analyzed by paired *t*-test and displayed as linked dot-plot of individual mice; crossbars mark the mean value of the data points. T, testosterone; DHT, 5α-dihydrotestosterone; n.s., not statistically significant.

Modulatory properties of the major male sex hormone testosterone are well established and likely contribute to sex dependent immunoregulation [28,29,40,41]. Androgens are supposed to act on T cells via membranous and nuclear androgen receptors [41,42]. Whereas coincubation with testosterone did not affect IL-22 release from male splenocytes, inhibition by the hormone was observed for female cells (Figure 3a). Inhibition of IL-22 was also observed when female splenocytes were co-incubated with 5α-dihydrotestosterone (DHT) (Figure 3b) which, in contrast to testosterone, cannot be further metabolized in aromatase-dependent fashion to become the principal female sex hormone estrogen [40,41]. Interestingly, splenocyte expressions of TNFα (Figure 3c) and IL-10 (Figure 3d) were both unaffected by testosterone or DHT.

IL-22 production by splenocytes was additionally investigated under the influence of Th17-promoting conditions. For this purpose, cells were polarized by cultivation in presence of αCD3, IL-23, IL-6, and transforming growth factor (TGF)-β (see Section 4). As shown in Figure 3e, production of IL-22 was significantly reduced (by 32.9%) under the influence of testosterone. Interestingly, IL-17 production was only marginally affected (reduction by 9.0%) in these same cultures (Figure 3f).

### 2.4. Androgen-Mediated Inhibition of IL-22 Expression in Murine T Lymphoma EL-4 Cells

**Figure 4:**
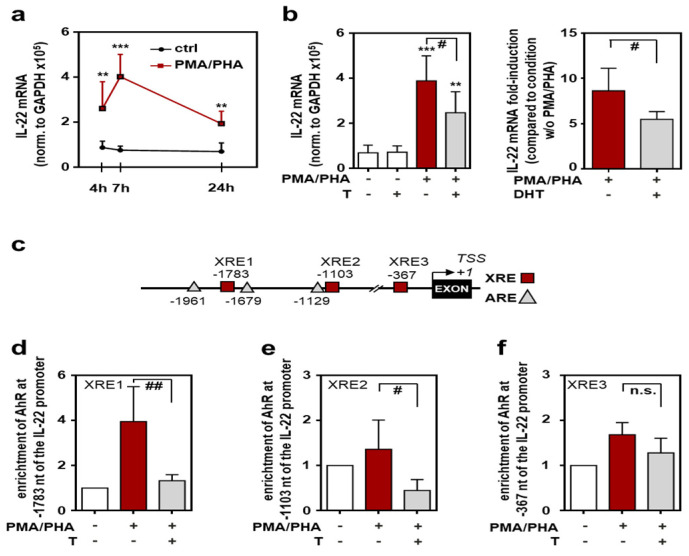
Androgens interfere with Ahr functions at the *il22* promoter: EL-4 cells were either kept as unstimulated ctrl or stimulated with PMA (100 ng/mL) and PHA (1 µg/mL) for the indicated time points (**a**), for 7 h (**b**), or for 3 h (**d–f**). Where specified, cells were pre-treated with testosterone (1 µM) or DHT (1 µM) for 15 days (**b**,**d**–**f**). All cultures were adjusted to a final concentration of 0.01% dimethyl sulfoxide (DMSO) (vehicle for PMA) and (if applicable) to 0.0014% methanol (vehicle for testosterone) or 0.029% methanol (vehicle for DHT). (**a**,**b**) mRNA expression for IL-22 was determined by real-time PCR, normalized to GAPDH and is shown as absolute values (**a**,**b**-left) or fold-induction compared to the condition without PMA/PHA (**b**-right) ((**a**,**b**-left): *n* = 6; (**b**-right), *n* = 5; ** *p* < 0.01, *** *p* < 0.001 versus the respective ctrl, # *p* < 0.05). (**c**) Schematic of XRE- and ARE-Sites within the first 2 kb of the murine IL-22 promoter. Numbers represent the distance from the putative *il22* transcriptional start site (TSS of NM_016971.2). (**d**–**f**) ChIP analysis was performed for detection of Ahr binding to the *il22* promoter region (**d**), −1783 bp/−1780 bp (XRE1); (**e**), −1103bp/−1100bp (XRE2); (**f**), −367 bp/−364 bp (XRE3)) (*n* = 5; # *p* < 0.05, ## *p* < 0.01). Statistical analysis: (**a**,**b**-right,**d**–**f**) raw data or fold-inductions/enrichments were analyzed by unpaired Student’s *t*-test; (**b**-left) raw data were analyzed by one-way ANOVA with post hoc Bonferroni correction; data are shown as means ± SD. T, testosterone; DHT, 5α-dihydrotestosterone; n.s., not statistically significant.

To study the regulation of murine IL-22 on a promoter basis, experiments were performed using T cell lymphoma EL-4 cells. These cells are principally sensitive to testosterone [43] and display significantly increased expression of IL-22 under the influence of phorbol 12-myristate 13-acetate (PMA)/phytohemagglutinin (PHA) (Figure 4a). Interestingly, PMA/PHA-induced IL-22 expression was only modestly affected by a temporary co-incubation with testosterone. Specifically, IL-22 mRNA expression (normalized to glyceraldehyd-3-phosphate-dehydrogenase (GAPDH)) was reduced by 18.0% (*n* = 6, *p* < 0.05, paired *t*-test; Supplementary Figure S3) under the influence of testosterone coincubation (at 1 μM). In contrast, after a 15 d period of testosterone conditioning, PMA/PHA-induced IL-22 was efficiently reduced (by 36.5%) in androgen-pretreated EL-4 cultures (Figure 4b, left panel). In contrast to IL-22, PMA/PHA-induced upregulation of TNFα was not affected by testosterone conditioning in those same samples (3.4 ± 1.4-fold-induction versus 3.1 ± 1.2-fold-induction for PMA/PHA versus PMA/PHA plus testosterone, *n* = 6). Accordingly, we also observed that PMA/PHA-mediated IL-22 gene induction was significantly impaired after a 15 d DHT-conditioning period (Figure 4b, right panel).

Ahr is regarded to be of pivotal importance for efficient IL-22 production by diverse cell types, most important Th17, γδT cells, and type 3 innate lymphoid cells [44,45]. The murine *il22* promoter actually displays three distinct Ahr-binding xenobiotic responsive elements (XRE1-3) within 2 kB upstream of the putative *il22* transcriptional start site. Interestingly, two of these sites (XRE1 and XRE2) are located in the vicinity of androgen responsive elements (ARE) (Figure 4c). In a consecutive next step, chromatin immunoprecipitation (ChIP) analysis was performed to evaluate physical binding of Ahr to the *il22* promoter under control conditions or the influence of testosterone. Binding of Ahr to XRE1 (Figure 4d) and XRE2 (Figure 4e) elements, but not to the XRE3 element (Figure 4f), was significantly reduced after testosterone conditioning of EL-4 cells.

## 3. Discussion

IL-22 is a cytokine with remarkable tissue-protective properties that particularly apply to the liver [3,15,24]. F-652, an IL-22-based biopharmaceutical agent, was recently shown to be effective in a phase II clinical trial addressing patients with alcoholic hepatitis [46]. Given these striking biological characteristics and having in mind the prospects of personalized medicine [47], knowledge on the role of sexes in the regulation of IL-22 production appears to be essential. By analyzing APAP-induced ALI, herein we identified IL-22 as a sex-related cytokine. Specifically, as compared to their male counterparts, female mice displayed significantly enhanced IL-22 expression, which became apparent at local hepatic and systemic levels. Because female mice are far less sensitive to APAP-induced ALI, it is tempting to speculate that endogenous levels of IL-22 may contribute to this phenomenon, in addition to sex-dependent effects on levels of protective glutathione [30] or SH3 domain-binding protein-5 [31]. Although application of recombinant IL-22 is clearly protective [20,21,22], the role of endogenous IL-22 in APAP intoxication appears to be complex. Specifically, a previous report demonstrates disease aggravation not only in IL-22^-/-^ mice, but, surprisingly, also in mice deficient for the IL-22 opponent IL-22 binding protein [32]. Further studies are required to clarify this paradox.

Increased IL-22 expression levels in female mice may potentially be due to a generally amplified female inflammatory response, as previously observed in murine lupus [48]. However, female IL-22 upregulation as detected during APAP intoxication was not connected to a higher degree of inflammation and displayed specificity because hepatic expression of paradigmatic inflammatory surrogates, such as CXCL2 and TNFα, were similar between both sexes.

To study sex-related regulation of IL-22 at a cellular level, experiments were performed using murine splenocytes and T cell lymphoma EL-4 cells. Although data derived from leukemic cell lines must be carefully assessed, EL-4 cells were previously used to study molecular biology of IL-22 expression, thus representing a suitable/reproducible model system that enables gene characterization at the promoter level [49]. Splenocyte experiments confirmed an increased female propensity to produce IL-22 in an authentic composition of murine leukocytes. This observation was independent of the mode of stimulation, which represented activation of either innate (LPS) or adaptive (αCD3/CD28) immunity. Of note, it has been shown that innate inflammatory stimulation is capable of activating IL-22 production by (memory) CD4^+^/CD8^+^ T cells through action of cytokines such as IL-1 [39,50,51]. In these same female splenocyte cultures in which enhanced IL-22 production was evident, sex-dependent regulation of IL-10, IFNγ, and TNFα was lacking, an observation again indicating specificity of IL-22 regulation.

The key male hormone testosterone has been linked to immunomodulation [40,41]. Interestingly, testosterone or DHT inhibited IL-22 production by LPS-stimulated female splenocytes, but not that of IL-10 or TNFα. It is furthermore remarkable that IL-22 regulation by testosterone was only detected in female splenocytes but not in that of males. This observation suggests that in vivo exposure of male splenocytes to testosterone is capable of mediating regulatory effects during the succeeding period of ex vivo cell culture. Moreover, in a protocol of adaptive splenocyte Th17 differentiation, testosterone impaired female IL-22 production but only marginally reduced IL-17. This latter observation is at variance with a previous study reporting more efficient inhibition of IL-17 production by testosterone. That report did not investigate IL-22 [52]. The difference between the two studies is likely related to different protocols of Th17 differentiation. Specifically, here we used unfractionated splenocytes (not isolated splenic T cells [52]) in order to particularly allow an influence of innate immune cells during Th17 differentiation.

After demonstrating that testosterone is able to inhibit IL-22 production by female murine splenocytes, mechanistic studies were performed using EL-4 cells. In concurrence with aforementioned splenocyte data, testosterone and DHT significantly reduced IL-22 induction initiated by PMA/PHA. Because three putative androgen receptor-binding ARE elements are located in the vicinity of two Ahr-binding XRE elements (XRE1, XRE2) within the murine *il22* promoter (Figure 4c), we hypothesized that testosterone, possibly for steric reasons, may impair binding of Ahr to XRE1 and XRE2. In this context it is noteworthy that some cell culture conditions, such as cultivation in presence of fetal calf serum (FCS) (as performed herein), can support Ahr activation [53]. Moreover, general T cell activation mediates functional Ahr signaling even in the absence of other exogenous stimuli, which boosts the associated gene expression, including that of IL-22 [54,55]. ChIP analysis revealed significantly reduced binding of Ahr to XRE1 and XRE2 (but not to XRE3) under the influence of testosterone, supporting our hypothesis that binding of androgen receptors to ARE sites within the *il22* promotor may abrogate pro-transcriptional effects of Ahr signaling at this specific gene locus. Herein, we focused on the well-controlled murine system. Although the human *IL22* gene actually displays an (imperfect) XRE site at -1797 bp [NCBI Reference Sequence: NC_000012.12 with a putative TSS at 68.248.242 (nucleotide A from NM_020525.5); this TSS position is defined herein as +1] along with an adjacent ARE site (within a distance of 17 bp), large studies are required to clarify whether IL-22 expression is similarly sex related in humans. This is in particular the case as IL-22 production in humans not only is supposed to be determined by genetic parameters such as gene polymorphisms [56], but also affected by age [57] and lifestyle conditions such as smoking [58], and thus displays considerable interindividual variability.

Taken together, in vivo and cell culture data suggest that the female murine immune system, compared to the male counterpart, is capable of generating significantly higher amounts of IL-22, a cytokine that exerts protective functions at biological barriers. Furthermore, mechanistic data propose that direct actions of the androgen receptor at the *il22* locus are responsible for curbing IL-22 production in males. The data presented emphasize the necessity to factor in sex-dependent effects when using mice to model pathophysiology affected by IL-22.

## 4. Materials and Methods

### 4.1. Reagents

Agonistic rat anti-murine CD3 (clone 17A2) and hamster anti-murine CD28 (clone 37.51) monoclonal antibodies were purchased from BioLegend (San Diego, CA, USA). APAP and LPS (O55:B5, TLR grade) were obtained from Sigma-Aldrich (Taufkirchen, Germany). Testosterone (T) and DHT were obtained from Merck/Millipore (Darmstadt, Germany), PMA from Enzo Life Sciences (Lörrach, Germany), and PHA-M from Roche (Mannheim, Germany). Murine IL-6 was obtained from Peprotech Inc. (Frankfurt, Germany). Murine IL-23 and TGF-β were from R&D Systems (Wiesbaden, Germany).

### 4.2. Murine Model of APAP-Induced ALI

Experiments using male and female 9–10 week old (mean age: 65 days) C57BL/6J mice (MfD Diagnostics, Wendelsheim, Germany) were performed in compliance with the recommendations of the Animal Protection Agency of the Federal State of Hesse (Regierungspräsidium Darmstadt, Germany). The protocol was approved by the Regierungspräsidium Darmstadt (FU/1253). Overnight (10 h) fasted male/female mice underwent APAP intoxication as previously described [20] using a dosage of 300 mg/kg APAP. Briefly, fasted mice obtained i.p. injections of either warm 0.9% NaCl (B. Braun, Melsungen, Germany) or 300 mg/kg APAP (dissolved in warm 0.9% NaCl). Mice that obtained NaCl-only are referred to as control mice (ctrl). During the experiment, all mice had access to food and water ad libitum. After 6 or 24 h, the experiment was terminated and mice were sacrificed upon isoflurane (Abbvie, Ludwigshafen, Germany) anesthesia. Blood was taken from the retroorbital venous plexus and generated serum was stored at −80 °C. For processing of hepatic specimens, livers were perfused with PBS. In order to prepare for histological analysis, specimens were subsequently treated overnight with 4.5% buffered formalin. For analysis of mRNA expression levels, snap frozen liver samples were stored at −80 °C.

### 4.3. Analysis of ALI by Histology and Determination of Serum ALT Activity

Formalin-treated tissue specimens were embedded in paraffin. Thereafter, liver sections (4 μm) were generated and stained using H&E. Liver injury was quantified by analysis of serum ALT activity according to the manufacturer’s instructions (Reflotron; Roche Diagnostics, Mannheim, Germany).

### 4.4. Isolation and Cultivation of Murine Splenocytes

Spleens of male and female 9–11 week old (mean age: 70 days) C57BL/6J mice (MFD-Diagnostics GmbH) were excised and transferred to 5 mL ice-cold RPMI 1640 (Thermo Fisher Scientific, Langenselbold, Germany). Tissue was disintegrated over a nylon cell strainer (70 μm; BD Biosciences, Heidelberg, Germany). Cell suspensions were centrifuged at 500 g for 5 min at 4 °C and resuspended in 2 mL 0.83% NH_4_Cl for 2 min at room temperature. Red blood cell lysis was stopped by adding 10 mL cold RPMI 1640. Splenocytes were collected by centrifugation, washed in RPMI 1640, and resuspended in RPMI 1640 supplemented with 10% heat-inactivated FCS, 100 U/mL penicillin, and 100 μg/mL streptomycin (Thermo Fischer Scientific). A quantity of 6 × 10^6^ cells was seeded on 12 well polystyrene plates (Greiner, Frickenhausen, Germany) in 1 mL culture medium and cultured at 37 °C and 5% CO_2_. Splenocytes were stimulated as indicated in the figure legends.

### 4.5. Th17 Polarization Performed in Splenocytes

Splenocytes were resuspended in RPMI 1640 supplemented with 10% heat-inactivated FCS, 100 μg/mL streptomycin, and 100 U/mL penicillin (Thermo Fisher Scientific) and cultivated in six well polystyrene plates (Greiner) that had been pre-coated overnight with aforementioned agonistic anti-murine CD3 antibody (BioLegend, 4 μg/mL) at 37 °C and 5% CO_2_. Splenocytes were maintained (3 × 10^6^ cells in 1 mL of aforementioned culture medium; six-well polystyrene plates) in murine IL-23 (6 ng/mL), IL-6 (20 ng/mL) and TGFβ (3 ng/mL) in order to support Th17 differentiation (with or without testosterone—as indicated in the figure legend). Supernatants were analyzed for IL-17 and IL-22 production after a 4 day cultivation period.

### 4.6. Cultivation of EL-4 T Cell Lymphoma Cells

Murine EL-4 T-cell lymphoma cells (ATCC-TIB-39) were purchased from LGC Standards (Wesel, Germany). Cells were maintained in RPMI 1640 supplemented with 100 U/mL penicillin, 100 μg/mL streptomycin, and 10% heat-inactivated FCS (Thermo Fisher Scientific) at 37 °C and 5% CO_2_. For all experiments, 2.5 × 10^6^ cells were seeded on six well polystyrene plates in 1 mL of the aforementioned culture medium and stimulated as indicated in the figure legends. For a period of 15 days pre-incubation/conditioning with testosterone or DHT (at 1 μM), medium (including testosterone/DHT or vehicle) was changed every other day; control cells were maintained upon methanol at 0.0014% (vehicle for testosterone) or methanol at 0.029% (vehicle for DHT).

### 4.7. Cytokine Release Analyzed by Enzyme-Linked Immunosorbent Assay (ELISA)

Cell culture media or serum concentration of murine IL-22 (splenocyte experiments, DuoSet; serum determinations, Quantikine assay; both R&D Systems), IL-10, IFNγ (both DuoSet, R&D-Systems), IL-17A (denoted as IL-17 throughout the manuscript), and TNFα (both Thermo Fisher Scientific) were determined by ELISA according to the manufacturers’ instructions.

### 4.8. Detection of Cytokine and Glyceraldehyd-3-Phosphate-Dehydrogenase (GAPDH) mRNA by Realtime Polymerase Chain Reaction (PCR)

Total RNA, isolated by Tri-Reagent (Sigma-Aldrich) was transcribed using random hexameric primers (Qiagen, Hilden, Germany) and Moloney virus reverse transcriptase (Thermo Fisher Scientific, Langenselbold, Germany) according to the manufacturers’ instructions. During realtime PCR, changes in fluorescence were caused by the Taq polymerase degrading the probe that contains a fluorescent dye (GAPDH: VIC, all others: FAM; Thermo Fisher Scientific). Pre-developed reagents (Thermo Fisher Scientific) used for analysis of murine mRNA expression: GAPDH (4352339E), CXCL2 (Mm00436450_m1), IL-22 (Mm00444241_m1), TNFα (Mm00443258_m1), IL-22RA1 (Mm01192943_m1), IL-6 (Mm00446190_m1), IL-20 (Mm00445341_m1), IL-24 (Mm00474102_m1), and Ahr (Mm00478932_m1). Assay-mix was from Nippon Genetics (Düren, Germany). Realtime PCR was run according to the manufacturers’ instructions using QuantStudio 3 (Thermo Fisher Scientific). For analysis of IL-22 mRNA in liver tissue: one initial step at 95 °C (2 min) followed by 45 cycles at 95 °C (5 s) and 62 °C (30 s). For analysis of all other target mRNA species in liver, splenocytes, and EL-4 cells (and for IL-22 mRNA in splenocytes and EL-4 cells): one initial step at 95 °C (2 min) followed by 40 cycles at 95 °C (5 s) and 62 °C (30 s). Detection of the dequenched probe, calculation of threshold cycles (Ct values), and data analysis was performed using the Sequence Detector software. Relative changes in mRNA expression compared to unstimulated control and normalized to GAPDH were quantified by the 2^−ddCt^ method or were shown as 2^−dCT^ (expression relative to GAPDH, absolute values).

### 4.9. ChIP and Associated Realtime PCR

ChIP analysis of Ahr binding to XRE within the murine *il22* promoter was performed using EL-4 cells and by adapting a previously described protocol [59]. The *il22* promoter region examined is located on chromosome 10 of the Mus musculus strain C57BL/6J (NCBI Reference Sequence: NC_000076.7) with a putative TSS at 118.040.870 (nucleotide C from “NM_016971.2”). This TSS position is used as reference position and defined as +1 in order to indicate locations within the *il22* promoter. Sequence analysis of murine/human *il22/IL22* gene was performed by MatInspector (Genomatix, Munich, Germany). For ChIP analysis, 2 μg of an IgG control (Cell Signal, Frankfurt, Germany; clone E1D5H) or a mouse monoclonal anti-murine Ahr antibody (Thermo Fisher Scientific, clone RPT9) were used. In order to amplify regions of the *il22* promoter containing XRE1 (−1783 bp/−1780 bp), XRE2 (−1103 bp/−1100 bp), and XRE3 (−367 bp/−364 bp) the indicated primers and probes were used for realtime PCR analysis. XRE1: forward primer 5′-CTAATGACTGGAGTCCGCTGC-3, reverse primer 5’-AATGCAGAAAGTTGAAAGGTGGCC-3’; probe 5’-CTTGAGCACGCTCTCCTCTG-3’. XRE2: forward primer 5’-GATCTCAATTAGCTGAGGGGAG-3’, reverse primer 5’-TACACTGAATCCCAGATAGCACC-3’; probe 5’-CGCGTTCTAGTCTAGATGTAGG-3’. XRE3: forward primer 5’-GGGAGATCAAAGGCTGCTCTA-3’, reverse primer, 5’-CCACCTTGAGAGATGGGAAGT-3’; probe 5’-GCAAAAGCACCTTGTTGGCCC-3’. Assay-mix was from Nippon Genetics. Realtime PCR was performed on a QuantStudio 3 (Thermo Fisher Scientific). One initial step at 95 °C (2 min), followed by 40 cycles at 95 °C (5 s) and 62 °C (30 s). Detection of the dequenched probe, calculation of threshold cycles (Ct values), and data analysis was performed by the Sequence Detector software. Enrichment of promoter DNA was calculated by the 2^−dCt^ method.

### 4.10. Statistics

Data were first evaluated by the D‘Agostino and Pearson test for parametric distribution. In case of ‘*n* < 8′, the Kolmogorov–Smirnov test was used. ‘n-numbers’ throughout the manuscript either refer to individual mice (Figure 1, Figure 2 and Figure 3, Supplementary Figures S1 and S2) or to independently performed experiments using EL-4 cells (Figure 4, Supplementary Figure S3). For two groups, raw data or fold-inductions/enrichments were analyzed by unpaired two-tailed Student’s *t*-test or paired (Figure 3e,f, Supplementary Figure S3) Student’s *t*-test, or by Mann–Whitney-U-test. For comparison of three or more groups, raw data were analyzed by one-way analysis of variance with post-hoc Bonferroni correction (ANOVA) or by Kruskal–Wallis with post-hoc Dunn’s test (Figure 1A). Potential outliers (Figure 2, Supplementary Figure S1) were not excluded from statistical analysis. Data are shown as means ± SEM or ± SD or as box-plots (with top and bottom margins referring to the 75th and 25th percentile, with whiskers depicting maximum and minimum values, with a horizontal line indicating the median, and with outliers identified as values beyond ± 1.5× IQR (Figure 2)) and presented as raw data, ng/mL, pg/mL, fold-induction, enrichment of promoter DNA, or as units/L. Differences were considered statistically significant if the *p*-value was below 0.05 (GraphPad Prism, La Jolla, CA, USA). Statistical information is specified in each figure legend.

## Supplementary Materials

The following are available online at https://www.mdpi.com/article/10.3390/ijms221910623/s1.
