# Supplementary material for: Acetaminophen-Induced Liver Injury Exposes Murine IL-22 as Sex-Related Gene Product
Source: Int J Mol Sci. 2021 Sep 30;22(19):10623. doi: 10.3390/ijms221910623 (PMC8509061; doi:10.3390/ijms221910623)
Supplement: Supplementary file 1 [file ijms-22-10623-s001.zip › Supplementary Figures/Suppl Figure S1_1350533.pptx]

## Slide 1
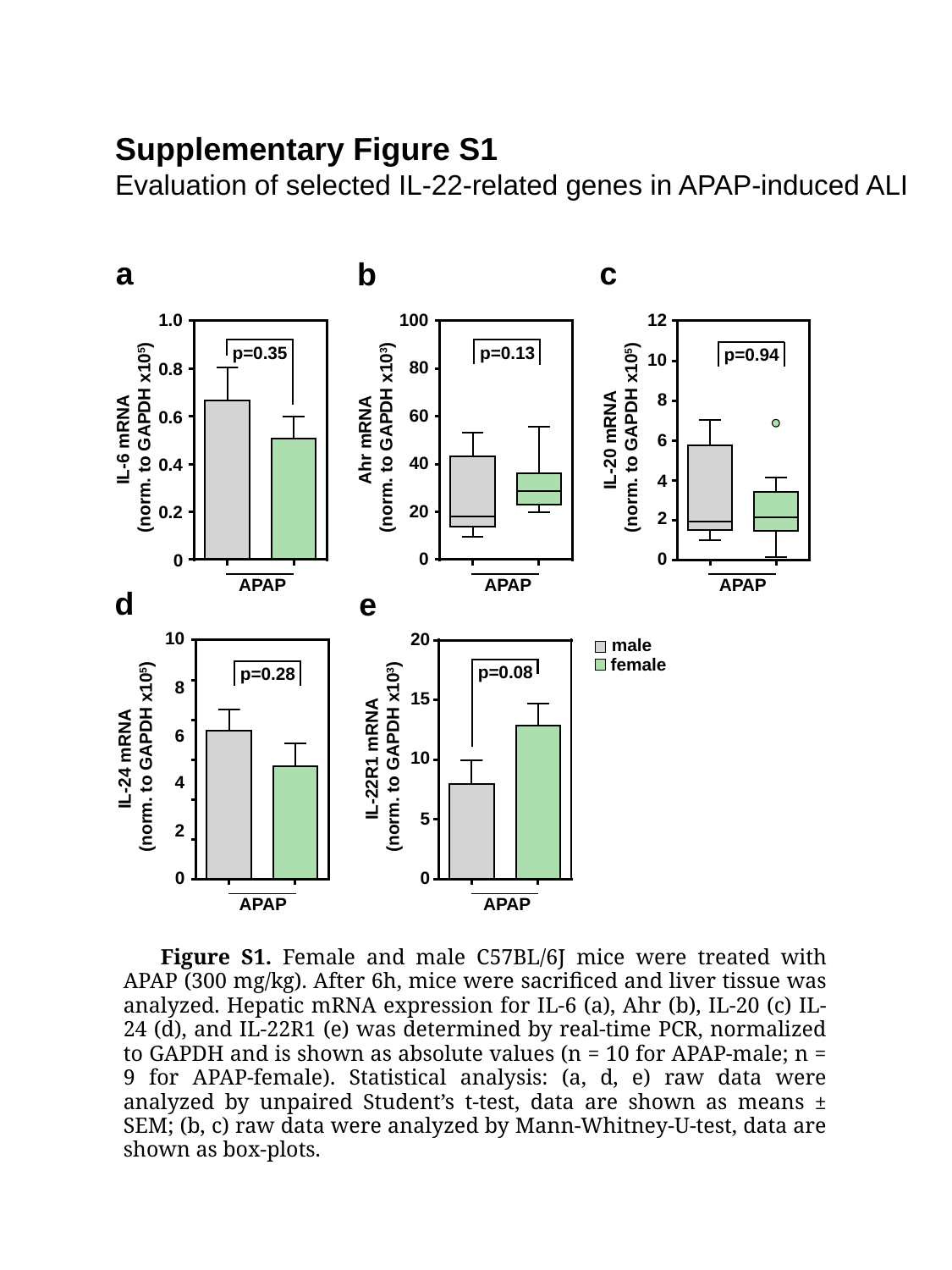

Supplementary Figure S1
Evaluation of selected IL-22-related genes in APAP-induced ALI
a
c
b
1.0
100
p=0.13
p=0.35
80
0.8
60
0.6
IL-6 mRNA
 (norm. to GAPDH x105)
Ahr mRNA
 (norm. to GAPDH x103)
40
0.4
20
0.2
0
0
APAP
APAP
12
p=0.94
10
8
IL-20 mRNA
 (norm. to GAPDH x105)
6
4
2
0
APAP
d
e
10
p=0.28
8
6
IL-24 mRNA
 (norm. to GAPDH x105)
4
2
0
APAP
20
p=0.08
15
IL-22R1 mRNA
 (norm. to GAPDH x103)
10
5
0
APAP
male
female
Figure S1. Female and male C57BL/6J mice were treated with APAP (300 mg/kg). After 6h, mice were sacrificed and liver tissue was analyzed. Hepatic mRNA expression for IL-6 (a), Ahr (b), IL-20 (c) IL-24 (d), and IL-22R1 (e) was determined by real-time PCR, normalized to GAPDH and is shown as absolute values (n = 10 for APAP-male; n = 9 for APAP-female). Statistical analysis: (a, d, e) raw data were analyzed by unpaired Student’s t-test, data are shown as means ± SEM; (b, c) raw data were analyzed by Mann-Whitney-U-test, data are shown as box-plots.
