# Supplementary material for: Acetaminophen-Induced Liver Injury Exposes Murine IL-22 as Sex-Related Gene Product
Source: Int J Mol Sci. 2021 Sep 30;22(19):10623. doi: 10.3390/ijms221910623 (PMC8509061; doi:10.3390/ijms221910623)
Supplement: Supplementary file 1 [file ijms-22-10623-s001.zip › Supplementary Figures/Suppl Figure S2_1350533.pptx]

## Slide 1
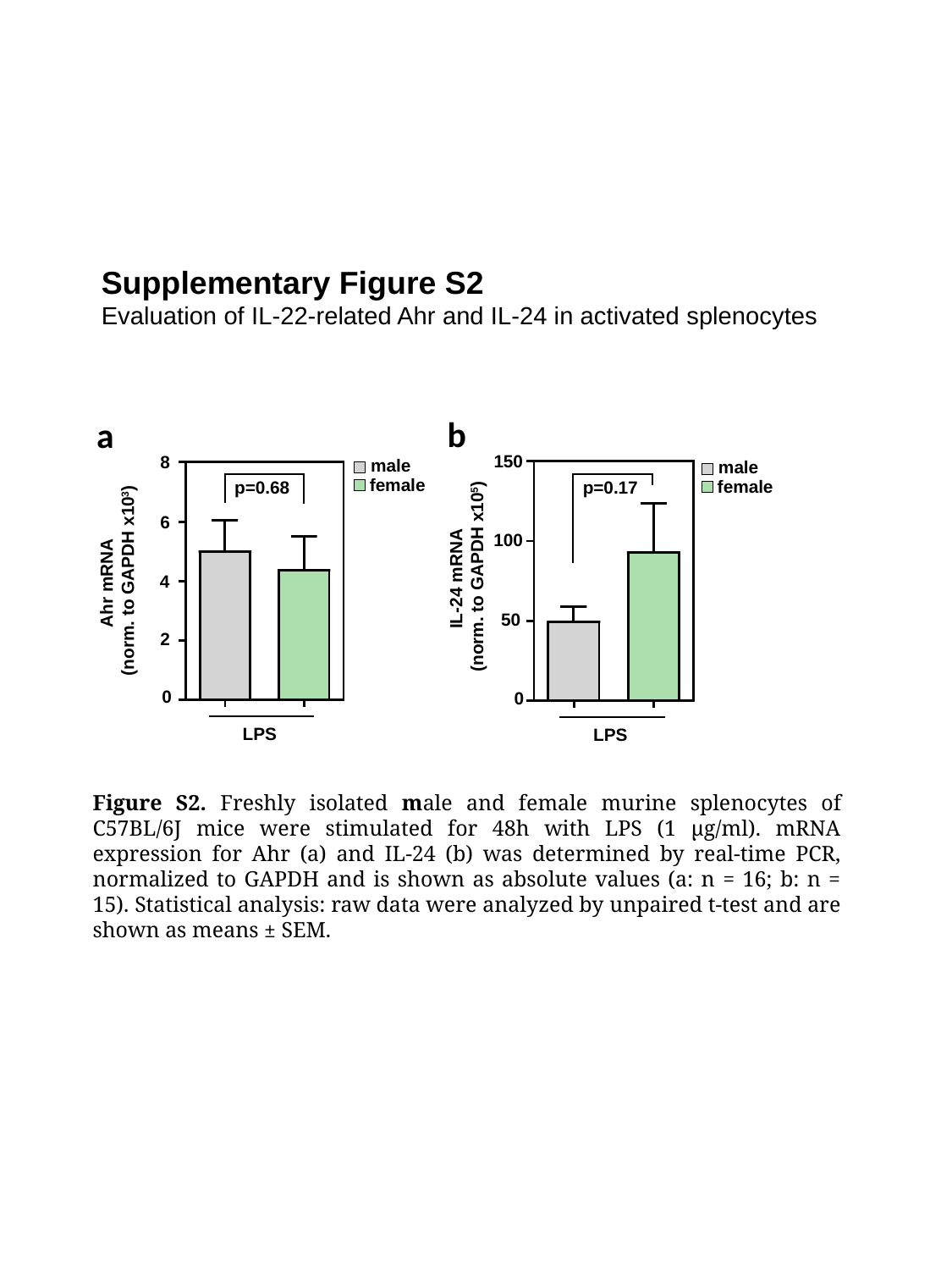

Supplementary Figure S2
Evaluation of IL-22-related Ahr and IL-24 in activated splenocytes
b
a
150
8
male
male
female
female
p=0.68
p=0.17
6
100
IL-24 mRNA
 (norm. to GAPDH x105)
Ahr mRNA
 (norm. to GAPDH x103)
4
50
2
0
0
LPS
LPS
Figure S2. Freshly isolated male and female murine splenocytes of C57BL/6J mice were stimulated for 48h with LPS (1 µg/ml). mRNA expression for Ahr (a) and IL-24 (b) was determined by real-time PCR, normalized to GAPDH and is shown as absolute values (a: n = 16; b: n = 15). Statistical analysis: raw data were analyzed by unpaired t-test and are shown as means ± SEM.
