# Supplementary material for: Acetaminophen-Induced Liver Injury Exposes Murine IL-22 as Sex-Related Gene Product
Source: Int J Mol Sci. 2021 Sep 30;22(19):10623. doi: 10.3390/ijms221910623 (PMC8509061; doi:10.3390/ijms221910623)
Supplement: Supplementary file 1 [file ijms-22-10623-s001.zip › Supplementary Figures/Suppl Figure S3_1350533.pptx]

## Slide 1
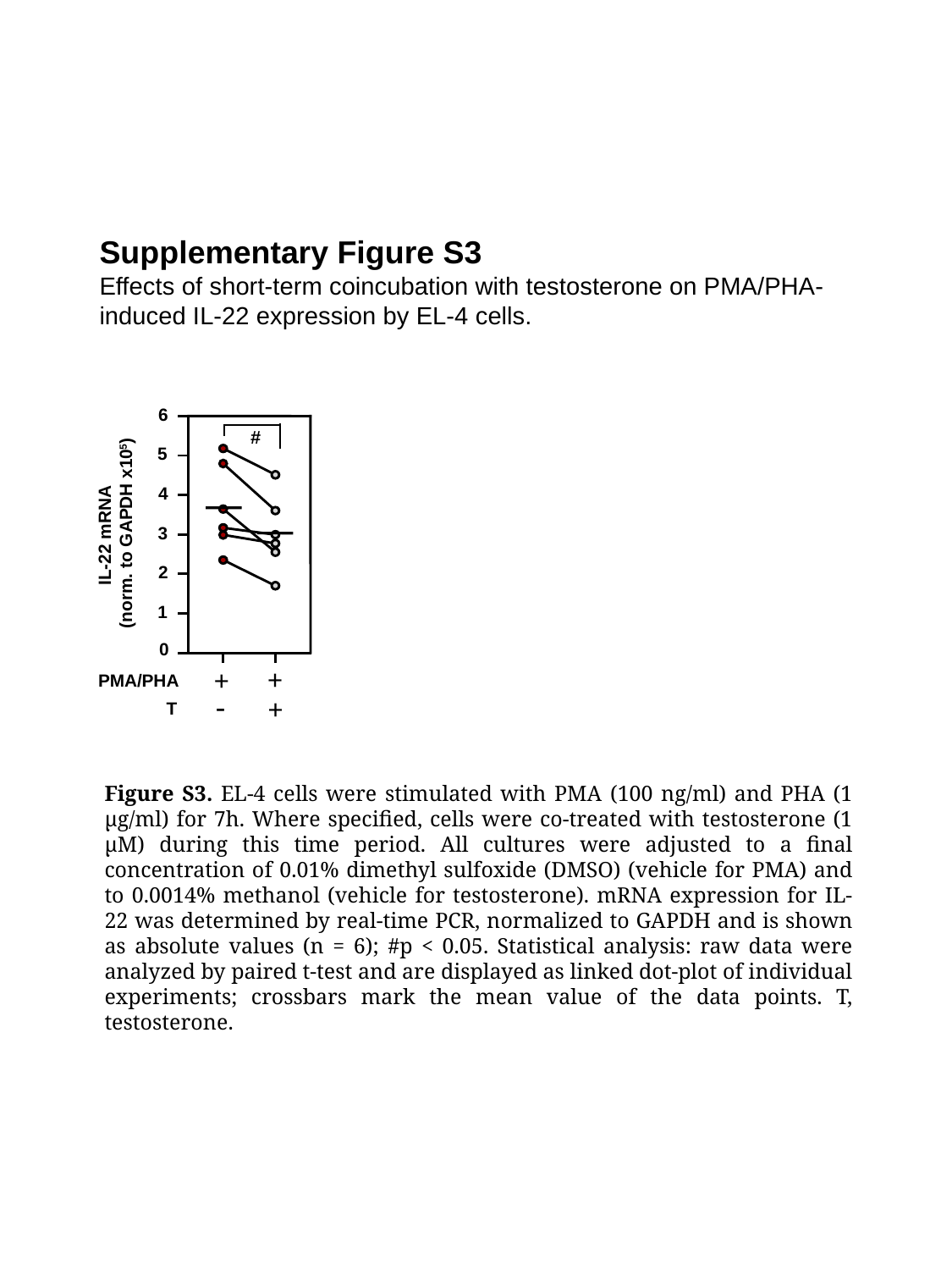

Supplementary Figure S3
Effects of short-term coincubation with testosterone on PMA/PHA-induced IL-22 expression by EL-4 cells.
6
#
5
4
IL-22 mRNA
 (norm. to GAPDH x105)
3
2
1
0
+
+
PMA/PHA
-
+
T
Figure S3. EL-4 cells were stimulated with PMA (100 ng/ml) and PHA (1 µg/ml) for 7h. Where specified, cells were co-treated with testosterone (1 µM) during this time period. All cultures were adjusted to a final concentration of 0.01% dimethyl sulfoxide (DMSO) (vehicle for PMA) and to 0.0014% methanol (vehicle for testosterone). mRNA expression for IL-22 was determined by real-time PCR, normalized to GAPDH and is shown as absolute values (n = 6); #p < 0.05. Statistical analysis: raw data were analyzed by paired t-test and are displayed as linked dot-plot of individual experiments; crossbars mark the mean value of the data points. T, testosterone.
